# Using evolution as a guide to engineer kranz-type c4 photosynthesis

**DOI:** 10.3389/fpls.2013.00212

**Published:** 2013-07-01

**Authors:** Thomas L. Slewinski

**Affiliations:** Department of Plant Biology, Cornell UniversityIthaca, NY, USA

**Keywords:** Kranz anatomy, C_4_ photosynthesis, bundle sheath, endodermis, SCARECROW, SHORT-ROOT, phyllode theory, evolution

## Abstract

Kranz-type C_4_ photosynthesis has independently and rapidly evolved over 60 times to dramatically increase radiation use efficiency in both monocots and eudicots. Indeed, it is one of the most exceptional examples of convergent evolution in the history of life. The repeated and rapid evolution of Kranz-type C_4_ suggests that it may be a derivative of a conserved developmental pathway that is present in all angiosperms. Here, I argue that the Kranz-type C_4_ photosynthetic system is an extension of the endodermis/starch sheath, that is normally only found in the roots and stems, into photosynthetic structures such as leaves. Support for this hypothesis was recently provided by a study that showed that the same genetic pathway that gives rise to the endodermis in roots, the SCARECROW/SHORT-ROOT radial patterning system, also regulates the development of Kranz anatomy and C_4_ physiology in leaves. This new hypothesis for the evolution of Kranz-type C_4_ photosynthesis has opened new opportunities to explore the underlying genetic networks that regulate the development and physiology of C_4_ and provides new potential avenues for the engineering of the mechanism into C_3_ crops.

## THEORY AND DISCUSSION

A new revolution in agriculture is needed to keep pace with the demands of humanity in the next century (Fedoroff et al., 2010). More humans will be alive at one time than ever before in earth’s history. Human population has been on the same trajectory for decades, regardless of food availability (Fedoroff and Cohen, 1999). Until recently, food production was able to stay ahead of the overall needs of the population. The first green revolution provided enough food to avoid mass starvation at the time of its implementation, as well as a surplus to cope with the population increase in the last half century (Borlaug, 2007). But now, based on our current resource availability, agricultural productivity, and projected consumption rates, some suggest we will approach or surpass human carrying capacity on the planet (Borlaug, 2002). The gap between agricultural surplus and human needs is narrowing fast, or has at this point in time, closed.

Crop breeding and biotechnology in the last century have altered plants in many drastic ways. Breeders were able to increase the harvest index, growth rate, biomass accumulation, disease and pest resistance, biotic and abiotic stress tolerance, and nutrient use efficiency, while also extending the climatic range of crops into previously unproductive regions (Fischer and Edmeades, 2010). However, the rate of improvement in these areas is still outpaced by human demands (Brown, 2012). Increases in actualized yields are becoming harder to achieve (Reynolds et al., 2011). Additionally, most beneficial traits that have been exploited to date also require a simultaneous increase in inputs.

One trait that has not significantly changed is the efficiency of photosynthesis (Reynolds et al., 2011). The maximum productive output per unit photosynthetic area, i.e., radiation use efficiency (RUE), has remained steady throughout domestication and selective breeding of most crops (Reynolds et al., 2009). If this previously recalcitrant trait could be modified, it could open up a new avenue of crop improvement. Now with the use of biotechnology, it has been suggested that increasing the RUE in C_3_ crops, by introducing the C_4_ photosynthetic mechanism, could be an effective way to increase yields by boosting productivity per unit area of land as well as reducing the amount of water and nitrogen used in achieving those yields (Hibberd et al., 2008; von Caemmerer et al., 2012). Integrating C_4_ photosynthesis into C_3_ crops may become even more necessary if climatic changes continue along the current trends (Sage and Zhu, 2011). For example, a recent report of soybean production in the Midwest of the USA revealed that the predicted fertilization effect of increased CO_2_ in the future may be negated by the increasing temperatures predicted for the coming decades (Ruiz-Vera et al., 2013). Thus, the one positive aspect of increased anthropogenic CO_2_ emissions that has been argued by many scientists may be undermined by the broader impacts of climate change.

Kranz-type C_4_ photosynthesis is one mechanism that plants have repeatedly and rapidly evolved to dramatically increase RUE and stress tolerance in hot and dry environments by reducing the rate of photorespiration in the carbon fixation process (for reviews and discussion on the biochemistry of C_4_ photosynthesis, see Langdale, 2011; Wang et al., 2011). This adaption has become even more effective in recent planetary history when carbon dioxide levels declined and oxygen levels increased. Current conditions are a stark contrast to the environment in which photosynthesis and Rubisco, the enzyme that fixes CO_2_ for entry into the Calvin cycle, first evolved (Sage, 2004). Thus it can be argued that, the more ancient C_3_ mechanism is best adapted for an environment that no longer exists. In contrast, the C_4_ mechanism overcomes the inherent limitations of Rubisco by dividing the photosynthetic process into two cell types. These cells are arranged into concentric circles around veins that produce a wreath-like appearance known as Kranz anatomy (Langdale et al., 1989). The bundle sheath (BS) cells comprise the inner circle attached to the vein and are responsible for the key reductive step in photosynthesis, carried out by Rubisco (Sage and Zhu, 2011; Sage et al., 2012; von Caemmerer et al., 2012). The mesophyll (M) cells encircle the BS and are responsible for the initial CO_2_ fixation by phosphoenolpyruvate carboxylase to produce the 4-C compounds malate or aspartate (Furbank, 2011). Malate or aspartate then moves from the M to the BS cells through plasmodesmata where CO_2_ is then released and then re-fixed by Rubisco. This process concentrates CO_2_ around Rubisco while also excluding oxygen in the BS, thus eliminated the metabolic drag of photorespiration that is common in C_3_ photosynthesis (Sage et al., 2012).

Previously, it was proposed that there are five major phases of morphological and physiological adaptations that plants undergo in the evolutionary trajectory toward C_4_ photosynthesis (Sage et al., 2012). The first proposed step is preconditioning which includes increasing vein density and possible gene duplication. Second is modification of BS cells. This includes cell enlargement, production of more organelles, and altered localization of the chloroplasts and mitochondria. M cell volume is also reduced during this transition. Together these changes lead to a “Proto-Kranz” condition. Third is the installation of the basic photorespiratory CO_2_ pump which includes the reduction of the M:BS cell ratio, localization of the C_3_ cycle to the BS and activation of the basic C_2_ system. Fourth is the enhancement of C_4_ CO_2_ metabolic capture and pump cycle within the M cells, which includes up-regulation and M-specific expression of phosphoenolpyruvate carboxylase. Finally, in the optimization phase, anatomy and biochemistry are fine tuned to exploit the full efficiency of the C_4_ mechanism (Sage, 2004).

However, there are reasons to suggest that the evolutionary progression toward the C_4_ state has been rapid. Kranz-type C_4_ has independently evolved over 60 times, occurring throughout the angiosperms in both monocots and eudicots (Sage et al., 2011). Indeed, it is one of the most exceptional examples of convergent evolution in the history of life. Astonishingly, Kranz-type C_4_ appears almost “fully formed” in each of the evolutionary events where it has arisen (Langdale, 2011). There is little evidence that is a slow evolutionary progression toward the C_4_ state as C_3_–C_4_ intermediates are lacking for most of the extant C_4_ species. Many C_4_ species also have closely related C_3_ relatives suggesting recent and rapid appearance of the C_4_ syndrome within some families of plants (Sage et al., 2011). Indeed, Kranz-type C_4_ is a classic example of Goldschmidt’s “Hopeful Monsters” (Goldschmidt, 1933), in which spontaneous complexity rapidly appears in some branches of life, and cannot be easily explained in the Darwinian model of evolution. However, I interpret the repeated and rapid evolution of complete Kranz-type C_4_ differently. This “fully formed” phenomenon (Langdale, 2011) suggests that only simple changes in some of the innate genetic programs are required in order for C_4_ to arise from a C_3_ background (Westhoff and Gowik, 2010). Therefore, it is reasonable to hypothesize that Kranz-type C_4_ may be a modification or extension of a conserved morphogenetic pathway that is inherent to all of the angiosperms.

What conserved tissue or genetic program in C_3_ plants could give rise to such a complex mechanism as Kranz-type C_4_ photosynthesis? If we take the view that cells such as the BS are derived from other cells that are already programmed with many of the underlying C_4_ biochemical programs, it is reasonable to hypothesize that the BS cells themselves confer the underlying properties of the C_4_ mechanism. The reasoning behind this hypothesis is that all living cells are programed with a specific “identity” that is determined at the time when ground meristem cells initiate differentiation. For example, cells that create the boundary between the outside environment and the internal organs all have a shared epidermis “identity.” They may have various characteristics depending on their location on the plant and specific function, but all share similar morphological and physiological properties as well as underlying developmental and genetic programs. Thus, root epidermal cells share identity with leaf or stem epidermal cells. Therefore, it is possible that C_4_ BS cells may share a similar identity with cells elsewhere in the plant. In the case of Kranz-type C_4_ cells, this shared identity may confer the underlying programs needed to establish or precondition the C_4_ metabolic mechanism within in the context of photosynthetic tissues (Slewinski et al., 2012).

What other cells within all angiosperms could be similar to C_4_ BS cells? Katherine Esau may have already answered this question when she published her anatomical surveys in the 1940s and 1950s – before C_4_ was discovered (Esau, 1953). She described some atypical species of plants that had “starch sheaths” within the photosynthetic leaf blades. She also described all BS tissue in leaves as having properties of endodermal tissue. When we look back on these observations we find something striking. Many of the atypical plants that Esau (1953) described as having “starch sheaths” in the photosynthetic leaf blades, turned out to be Kranz-type C_4_ plants such as maize and sorghum. Indeed, we now know that the Kranz C_4_ BS in leaves share similarities with endodermal tissues in petioles, stems, and roots (Nelson and Dengler, 1997; Slewinski et al., 2012). In all of these tissues, the endodermis is comprised of a single cell layer that surrounds the vasculature, has suberized cell walls, and displays polar expression of the pin-formed (PIN) effluxors, which conduct auxin through this cell layer (Slewinski et al., 2012). Based on Esau’s detailed observations of leaf anatomy and a plethora of recent reports on C_4_ physiology and development, I present a new hypothesis for the rapid and repeated evolution of C_4_ photosynthesis in the angiosperms.

## HYPOTHESIS

The Kranz-type C_4_ photosynthetic mechanism arises when the endodermal/starch sheath program extends into photosynthetic structures, such as leaves, where it is normally repressed or underdeveloped. This leads to a synergistic interaction which can produce the novel C_4_ pathway from underlying components of both the C_3_ photosynthetic program and anatomical and metabolic features of the endodermis/starch sheath.

**FIGURE 1:**
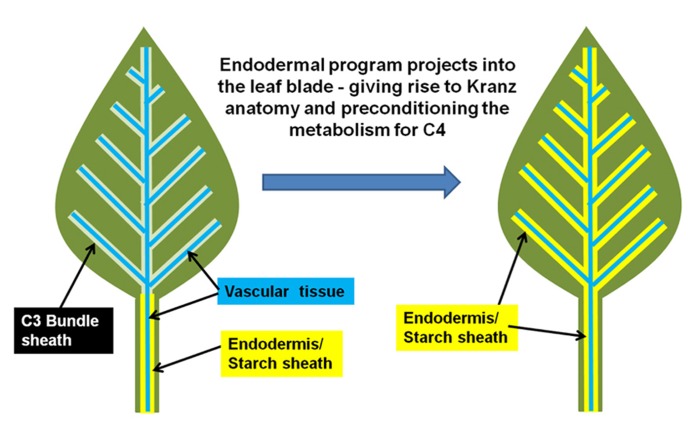
** Model for the evolution of Kranz-type C_4_ in dicots.** A normal C_3_ dicot leaf is represented on the right. The endodermis/starch sheath (yellow) is present on the vasculature (blue) of the petiole and lower leaf zone, but is absent in the leaf blade/upper leaf zone. The hypothesized shift from C_3_ to Kranz-type C_4_ may arise when the endodermal/starch sheath program extends out from the petiole/lower leaf zone into the leaf blade.

In other words, this suggests that the Kranz-type C_4_ mechanism is the context-specific manifestation of the endodermis in a photosynthetic tissue. The C_4_ condition arises when the endodermis projects into the photosynthetic tissues, which also extends the properties of the endodermal/starch sheath program from stem and petiole into the leaf (Slewinski et al., 2012). A schematic of this hypothesis is presented in **Figure 1**. Thus, the inherent physiology of the endodermis may integrate into the photosynthetic program, resulting in a new synergistic physiology, which we know as C_4_ photosynthesis.

In plants, the tissue in which a cell resides usually determines the cell’s function and physiological properties. This reasoning can be applied to the endodermis, which is a dynamic tissue that appears to have context-dependent functions. For example, in roots the endodermis encircles the vascular core of the root and acts as an internal barrier for solute transport from the cortex and epidermal cell layers that interact with the external soil environment (Alassimone et al., 2012). At the root tip, columella cells have different properties than their adjacent stem cells, which are also part of the endodermal tissue (Welch et al., 2007; Ogasawara et al., 2011). Along the root length, endodermal cells do not accumulate starch whereas the endodermis in the stem and petiole does, and is thus termed the “starch sheath” (Wysocka-Diller et al., 2000). The starch sheath usually extends along the vascular core(s) from the base of the shoot–root junction to the petiole–leaf blade junction. The starch-filled amyloplasts within these cells act as statoliths – providing gravity cues to the cells in a similar manner to the columella cells within the root tip (Morita et al., 2007). Within these cells, the amyloplasts display a polar localization at the base of the cell, in the direction of gravitational pull. Changes in amyloplast position in these cells trigger changes in auxin transport through the endodermal cell layer (Tanimoto et al., 2008). This results in differential cell expansion in the stem that properly orients the plant into the upright position, opposing the direction of gravity (Morita et al., 2007).

When comparing the many forms of endodermis that occur in plants, the C_4_ BS is most similar to that of the starch sheath in petioles and stems (Hibberd and Quick, 2002; Tanimoto et al., 2008). Interestingly, C_4_ chloroplasts are also similar to those found in the starch sheath of the stem and petiole in certain ways. First, the C_4_ BS chloroplasts preferentially accumulate starch when compared to M chloroplasts (Lunn and Furbank, 1997). Second, C_4_ chloroplasts usually have a fixed location in the cells, either on the cell surface adjacent to the vascular core or adjacent to the M (Morita et al., 2007). Third, in many C_4_ species, BS chloroplasts lack photosystem II and stacked thylakoid grana, similar to amyloplasts found in the starch sheath (Langdale, 2011). Although, there is great variation in all three of these characteristics in C_4_ species, similarities suggest that chloroplasts within the starch sheath and the C_4_ BS share components of their identity. Is it possible that chloroplasts in the C_4_ BS are essentially photosynthetic-amyloplasts, i.e., plastids of hybrid identity? This may explain why dimorphic chloroplasts are frequently associated with the C_4_ BS cells, because the BS cells have a mixed identity of both the starch sheath and photosynthetic cells. However, there is wide variation in BS chloroplast structure within the Kranz-type and single celled C_4_ species, suggesting that a range of amyloplast-like features are compatible with C_4_ BS, and that only a subset of associated starch sheath/amyloplasts mechanisms are required or sufficient to produce a functional C_4_ photosynthetic system.

**FIGURE 2:**
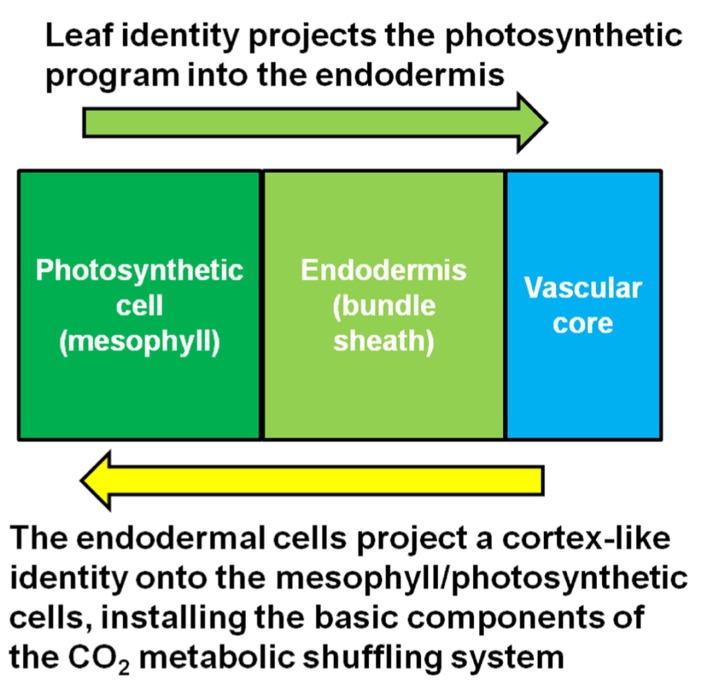
** Model for the synergistic interaction between the starch sheath and the photosynthetic cells.** Kranz-type C_4_ may arise when the photosynthetic system of the leaf integrates with the CO_2_ metabolic shuffling system usually found in the respiring tissues of the plant.

In an insightful paper by Hibberd and Quick (2002), it was shown that the starch sheath in aerial parts of the plant, especially petioles, is involved in internal CO_2_ recycling (Hibberd and Quick, 2002). Respiring tissues such as roots produce abundant CO_2_ as a waste product. However, not all of the CO_2_ is released into the soil environment that surrounds the roots (Bloemen et al., 2013). Much of the respired carbon migrates into the xylem stream that flows from the roots toward the leaves. A study using mature poplar trees shows that a significant portion of the respired carbon in roots eventually ends up re-fixed in the petioles at the base of leaves (Bloemen et al., 2013). This carbon is most likely in the form of malate (Hibberd and Quick, 2002), a neutral compound that does not impact pH like carbonic acid, which is produced when CO_2_ dissolves either in the cytosol or apoplastic water reserves which flow into the xylem stream. In *Arabidopsis*, tobacco and celery xylem-derived malate is re-assimilated in the photosynthetic endodermal/starch sheath cells that surround the vasculature within the petiole and leaf mid-vein (Hibberd and Quick, 2002). Most of this carbon ends up in starch during the day, then is mobilized and transported in the phloem in the form of sucrose to sink tissues at night. It is reasonable to hypothesize that this is the precursor mechanism that gives rise to CO_2_ metabolic shuffling in the C_4_ mechanism. In other words, C_4_ metabolic shuffling may be an extension of the internal CO_2_ recycling system. In the case of Kranz-type C_4_, this CO_2_ waste management system extends out from the petiole with the endodermal program, giving rise to both Kranz anatomy while also preconditioning the leaf tissue for intercellular CO_2_ metabolic shuffling. It is likely that once the full endodermal/starch sheath program extends into the leaf, the synergistic interaction between the photosynthetic cells and the endodermis initiates the C_4_ metabolic mechanism, schematically represented in **Figure 2**. The initial event may not generate a fully functional C_4_ mechanism immediately, but may give rise to the so called “C_3_–C_4_ intermediates” which possess the correct architecture, and have properties of both the C_3_ and C_4_ mechanisms. Further selection for the C_4_ mechanism may be required to suppress the remnants of the C_3_ photosynthetic pathway that are unneeded or redundant, while concurrently enhancing the more dominant features of the C_4_ metabolic pathway. This is not to say that C_3_–C_4_ intermediates always represent a transitional stage; they may be fully adapted in their current form in many cases (Sage et al., 2011).

It can be argued that selection against the C_4_/starch sheath physiological program in the C_3_–C_4_ intermediates is just as likely (Vicentini et al., 2008; Langdale, 2011). A full reversal of C_4_ to C_3_ is also possible and has already been reported in some of the C_3_ grasses (Vicentini et al., 2008). As a result, plants could arise that possess Kranz/C_4_-like anatomical features but with C_3_ photosynthetic metabolism. Another possibility is that C_3_–C_4_ intermediates, arising from either selection for or against the Kranz-type C_4_ pathway, could have some of the advantageous characteristics of full C_4_ plants in hot and dry environments (Sage et al., 2011). Thus, it can also be argued that development of C_4_-like traits can confer fitness on their own, implying that the C_3_–C_4_ intermediate state is an independent evolutionary trajectory (Langdale, 2011; Sage et al., 2011). Overall, this new view of C_4_ evolution suggests that only small changes are required to rapidly produce dramatic diversity in anatomy and physiology. This diversity is then subject to selection for or against the C_4_ mechanism based on the environmental pressures of the organism.

Selection for enzymatic cell specificity may also be necessary to increase the CO_2_ metabolic pump from the M to the BS, while also concurrently enriching Rubisco in the BS. Presumably, these two processes would evolve in parallel because sequestration of Rubisco to the BS without the CO_2_ pump would reduce carbon fixation in free air and lead to an evolutionary disadvantage. Interestingly, only phosphoenolpyruvate carboxylase is common to all of the decarboxylation types of C_4_ (Sage, 2004; Furbank, 2011). Extrapolation of the underlying endodermal physiology may occur differently with each independent evolutionary event – leading to variations in the CO_2_ metabolic pump, i.e., Nicotinamide adenine dinucleotide phosphate-malic enzyme (NADP-ME), Nicotinamide adenine dinucleotide-malic enzyme (NAD-ME), or phosphoenolpyruvate carboxykinase (PEPCK) types (Sage et al., 2011). However, recent evidence suggests that these three decarboxylation types may not be distinct, but are flexible depending on environmental and developmental conditions (Furbank, 2011; Pick et al., 2011). Within the grasses, switching of decarboxylation types within a species has been reported (Vicentini et al., 2008). However, if the three decarboxylation types are extrapolations of the underlying physiology of the endodermis/starch sheath program, then it is reasonable to hypothesize that each type is simply a dominate enzymatic pathway within a larger physiological context that includes subtle forms of the other two types. Section pressures on a recently evolved C_4_ species then determines which of the three decarboxylation types become dominant. The other pathways are most likely not eliminated in this selection but left in their original and more subtle “housekeeping” roles or suppressed to lower levels. Thus under this new hypothesis, significant plasticity and flexibility in the C_4_ mechanism would also be conferred by the underlying endodermal/starch sheath program.

**FIGURE 3:**
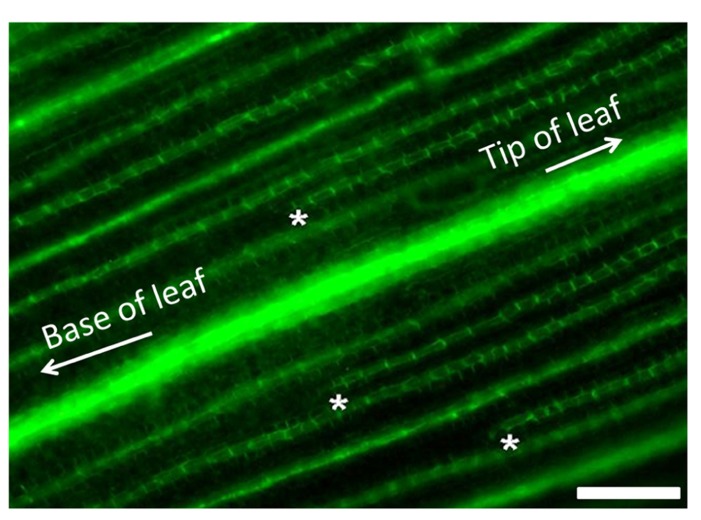
** Minor vein formation in developing maize leaves.** Visualization of PIN-YFP vascular marker in developing maize leaves showing minor vein formation. Minor veins initiate at the tip of the leaf and develop toward the base between the established large lateral and intermediate veins. Developing tips of minor veins are demarcated with white asterisks. Scale bar: 50 μm.

Following these arguments, it is important to also highlight that, in both roots and stems, the endodermis functions as a high-capacity auxin conducting tissue (Alassimone et al., 2012). In both C_3_ and C_4_ plants, vein patterning is regulated by auxin gradients generated by both synthesis and transport (Scarpella et al., 2010). Auxin produced in the epidermis drains toward preexisting veins within the developing tissues. When larger veins form, they also produce auxin gradients within the adjacent ground meristem tissue by depleting auxin from the surrounding cells. This creates an auxin minima that initiates the formation of smaller vein orders that form after the larger orders of veins have been established and are undergoing differentiation (Scarpella et al., 2010; Gardiner et al., 2011). The formation pattern of minor veins between established major and intermediate veins in maize is shown in **Figure 3**. The extension of the endodermal layer into the vascular tissue in the developing leaf may enhance the depletion of auxin from the ground tissue in C_4_ leaves when compared to developing C_3_ leaves. Under these assumptions, it is reasonable to hypothesize that the increased vein density observed in C_4_ plants is, at least in part, due to the increased auxin depletion associated with the developing endodermal layer. This would presumably create more or stronger auxin minima, thus initiating more minor veins.

Unlike non-Kranz species, each vein initiation confers the formation of entire Kranz units (vascular core, BS, and surrounding M cells; Nelson and Dengler, 1997). It is unlikely that veins can get closer than one vascular Kranz unit because of the nature of the underlying endodermal developmental program. In this model, the development and identity of the cells is determined by the signals generated from the vascular core – which first determines BS. BS cells then generate signals that determine M specification. Thus, the proximity and intercellular interactions from the endodermal developmental program may also confer a cortex-like identity on the already present photosynthetic M cells, modifying their development, architecture, and physiology. This may explain why C_4_ plants reduce M cell counts to the extent that they match BS cells, ultimately ending in a 1:1 ratio. In contrast, veins in C_3_ plants do not form such units. Rather they form in a pool of ground meristem cells – defining cells that will become part of the vasculature and excluding the cells that will give rise to the M. Thus, the C_3_ mode of vascular development leads to more variable numbers of M cells between vascular strands.

The shift in plasmodesmata density and specialization at the M–BS interface in the leaves of C_4_ plants may also be a pleiotropic effect of the endodermal program in the leaf. In other parts of the plant, the endodermis is coated with suberin and other hydrophobic compounds that create an apoplastic barrier that limits cell-to-cell flow of water and solutes through the cell wall (Geldner, 2013). Therefore most transport between the endodermal cells and the surrounding cortex or parenchyma cells is restricted to the symplastic route – through abundant plasmodesmata that connect the cytosolic domains of adjacent cells. Although the suberized apoplastic barrier is only sometimes associated with the C_4_ BS (Sage, 2004), increased intercellular symplastic transport between BS and M cells appears to be necessary for an efficient CO_2_ metabolic pump between M and BS cells. As with the other traits associated with C_4_ specialization mentioned above, the intercellular transport mechanism utilized by the C_4_ BS and M cells in the leaf may be an extension and modification of the system found in the endodermal tissue in the roots and stems.

In *Arabidopsis* the genes that underpin endodermis formation, *Scarecrow* (SCR) and *Short-root* (SHR), are expressed in roots, stems, and leaves (Wysocka-Diller et al., 2000; Gardiner et al., 2011). The *SHR* gene is expressed in cells within the vascular core (Helariutta et al., 2000), except for the phloem initial cells (Yu et al., 2010). The SHR protein moves out from the vascular core cells and activates the *Scr* gene within the cells that are in contact with the vascular core (Koizumi et al., 2012). SCR protein binds to SHR and sequesters the protein in the nucleus, preventing further movement (Wu and Gallagher, 2012). This mechanism deliminates a single cell layer as well as initiates the cascade of signals that establish endodermis identity. Thus, it is reasonable to hypothesize that if Kranz-type BS tissue is just an extension of the endodermal program, they should also be subject to mutations in the essential endodermal patterning and development genes SCR and SHR. Indeed, support for this reasoning was recently provided. It was shown that the maize ortholog of SCR plays a role in BS development in maize leaves (Slewinski et al., 2012). Mutations in the *ZmSCR* gene result in proliferation of BS cells, altered differentiation of BS chloroplasts, vein distortion, and reduction in minor vein formation and overall vein density. *zmscr* mutant plants also produce starch-less BS cells that closely resemble starch-less stem endodermal cells in the *shr* mutant of *Arabidopsis* called *endodermal amyloplasts less1* or *eal1* (Morita et al., 2007). In the *scr* mutant of maize, some of these starch-less cells also have altered plasmodesmata within the cell walls that separate the BS and M cells (Slewinski et al., 2012), suggesting that their specialization is also linked to the endodermal program. Thus, this provides for the first time, genetic evidence that the endodermal development pathway underlies C_4_ BS development. This study also suggests, though does not directly prove, that SHR also plays a critical role in the development of the BS and underlying metabolism in C_4_ plants.

**FIGURE 4:**
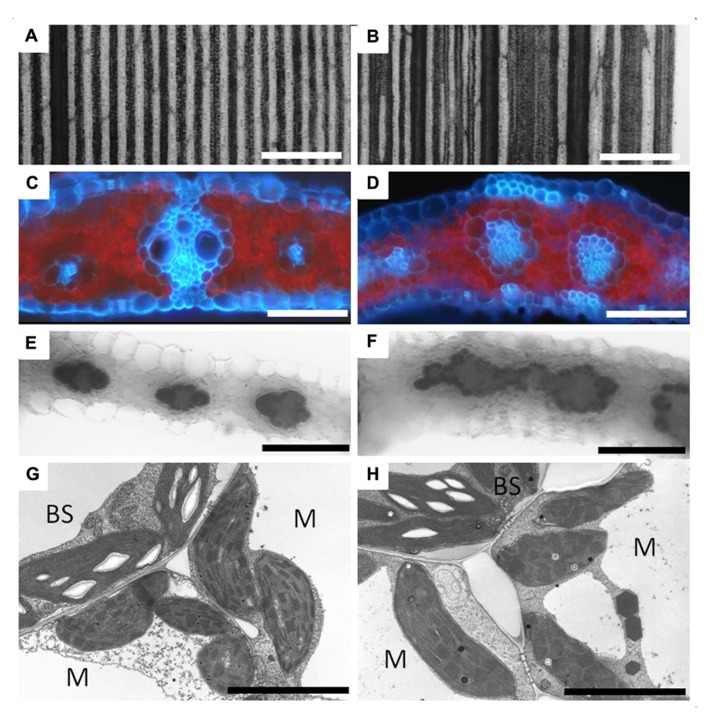
** Vascular development and bundle sheath formation in the *lsn* mutant of maize.** Panel showing wild type **(A,C,E,G)** and *lsn* mutant **(B,D,F,H)** maize leaf sections. **(A)** Section of iodine potassium iodide (IKI) stained wild type leaf showing regular and uniform vascular patterning. **(B)** Section of IKI stained *lsn* mutant leaf showing distorted vascular pattering. **(C)** Cross section of wild type leaf under UV light showing canonical Kranz anatomy (red represents chlorophyll autofluorescence, blue represents autofluorescence of the cell walls). **(D)** Cross section of the *lsn* mutant under UV light, showing distorted veins with internal vascular hypertrophy and irregular internal differentiation surrounded by single layers of bundle sheath and mesophyll cells. **(E,F)** Cross sections of IKI stained leaves, respectively, showing normal starch accumulation in the BS cells and absence of staining in the M cells. **(G,H)** Transmission electron micrographs of wild type **(G)** and *lsn* mutant **(H)** BS and M cells showing normal C_4_ plastid differentiation and identity in both. BS, bundle sheath cell, M, mesophyll cell, scale bars: **(A,B)** 600 μm; **(C–F)** 50 μm; **(G,H)** 5 μm.

Analysis of the *large scutellar node* (*lsn*) mutant of maize also supports the endodermal development model for C_4_ BS in leaves. The *lsn* mutant phenotype mimics the abnormalities observed when auxin transport inhibitors are applied to developing leaves (Landoni et al., 2000). These abnormalities include vein distortions, vascular hypertrophy, and disorganized vascular core structure (**Figures 4A,B**). What is interesting in the *lsn* mutant is the formation of normal BS and M, both structurally and physiologically, around the distorted vascular core in the leaves (**Figures 4C,D**; Landoni et al., 2000). *lsn* BS cells preferentially accumulate starch like wild type plants (**Figures 4E,F**), and both BS and M plastids appear normal in transmission electron microscopy (TEM) analysis (**Figures 4G,H**). This finding conflicts with the cell lineage models that have previously been proposed for the development of the C_4_ BS which suggested that BS and M cells arose from organized cell division patterns (Langdale et al., 1989; Sud and Dengler, 2000). However, BS formation in *lsn* more closely resembles the endodermis that surrounds distorted veins in *Arabidopsis* plants grown in the presence of auxin transport inhibitors (Wysocka-Diller et al., 2000), suggesting that organized and coordinated cell division is not essential for the development of Kranz anatomy. Although, analysis of *lsn* does fit within the framework of the endodermis/starch sheath developmental model (Helariutta et al., 2000). Additionally in *Arabidopsis*, the SCR::GFP construct is still only expressed in a single cell layer of endodermal cells when internal vascular hypertrophy or distortion occurs (Wysocka-Diller et al., 2000). This again suggests that both the development of the endodermis and C_4_ BS are regulated by a non-cell autonomous signal that radiates from the internal vascular core, most likely the SHR protein.

The role of SHR in recruiting C_4_ BS may also explain some of the diversity seen in Kranz anatomy. As noted above, phloem initial cells do not express the *Shr* gene and therefore must symplastically import the SHR protein to proceed through normal phloem differentiation (Yu et al., 2010). Thus, the developing phloem presumably acts as a SHR protein sink, rather than a source of the signal. Usually, the phloem is localized within the vascular core – completely surrounded by cells that produce the SHR protein signal (Vatén et al., 2011). However, there are some C_4_ species like *Atriplex rosea* which develop phloem bundles close to the edge of the vascular core in leaves (Dengler et al., 1995). In this species, the C_4_ BS only encircles part of the vein, and is absent where the phloem bundle protrudes from the vascular bundle. Indeed, many other C_4_ species show a similar arrangement, where C_4_ BS are either absent or converted to sclerenchyma cells in the regions adjacent to the phloem bundles (Edwards and Voznesenskaya, 2011). Therefore it is reasonable to hypothesize that the internal vascular structure influences the dynamics of non-cell autonomous developmental signaling of the endodermal/BS program which could lead to the wide variations in Kranz-type structures seen in many C_4_ species (Edwards and Voznesenskaya, 2011).

## ENGINEERING A NOVEL FUNCTION FOR A CONSERVED TISSUE

Esau (1953) suggested that all BS in angiosperms have some endodermis-like features. But the extent to which these endodermal features are manifested in the BS varies greatly. Therefore it is likely that in C_4_ plants, full Kranz anatomy arises when the underlying endodermal framework becomes enhanced – leading to the more dominant features that are associated with full endodermal/starch sheath identity. Following this reasoning, C_4_ physiology may also be a manifestation of sufficient endodermal/starch sheath identity extending into the leaf. This could also explain why intermediates between C_3_ and C_4_ are present in some species, and why it is perceived that anatomical shifts precede physiological changes in the evolutionary trajectory toward Kranz-type C_4_ (Sage et al., 2012).

How can this hypothesis for the evolution of Kranz-type C_4_ be used to transfer the syndrome to C_3_ plants? Again, we need to look at this issue in terms of tissue “identity” and it’s functions within a plant organ. Context-dependent tissue function is common in plants. For example, it is hypothesized that in angiosperms, petals are modified leaf structures – thus they are leaves in the context of a reproductive organ (von Goethe, 1790; Pelaz et al., 2001). Therefore, the same or similar genes that usually control leaf development also impact floral development. This hypothesis has been supported experimentally. For example, the ectopic over-expression of a set of transcription factors that usually give rise to petal identity, transform leaves into petal-like structures (Pelaz et al., 2001). The change from leaf to petal tissue also transforms plastid identity from photosynthetic chloroplasts into non-photosynthetic chromoplasts. This experiment shows that entire morphology and physiology of a leaf can be reprogrammed by modulating its “identity.” Most importantly, this was accomplished by altering the expression of a few transcription factors (Pelaz et al., 2001). Indeed, it seems that many aspects of tissue engineering through manipulation of developmental signaling, which is commonly used in the animal biology and medical community, may also be employed in plant anatomical and metabolic engineering.

This raises the question: can we use a similar approach, by directly manipulating tissue identity, to alter the physiology of C_3_ leaves to become more C_4_ like? If so, which transcription factors are the most likely targets for C_4_ engineering? From the hypothesis presented in this paper, the most obvious candidates are SHR and SCR. Surprisingly, in *Arabidopsis* both SHR and SCR proteins are already present in the cells that immediately surround the vasculature in the C_3_ *Arabidopsis* leaves (Wysocka-Diller et al., 2000; Gardiner et al., 2011), as they are in the developing root endodermis and in the stem starch sheath. This suggests that other interacting factors modulate the SCR/SHR complex in the C_3_ BS cells and that the endodermal program is most likely regulated on the protein level. These proposed SCR/SHR interacting proteins could either confer a specific cell identity (Welch et al., 2007; Ogasawara et al., 2011), or suppress the pathway as in the case of C_3_ leaves. The SCR/SHR pathway has been extensively studied in *Arabidopsis*, and yet very little has been reported on the function of these proteins in leaves (Wysocka-Diller et al., 2000; Yu et al., 2010; Gardiner et al., 2011; Ogasawara et al., 2011; Cui et al., 2012). From these data, it is reasonable to hypothesize that there may be a negative feedback loop to repress the endodermal developmental pathway in leaves. This may be why when either SHR or SCR are knocked out or over-expressed in *Arabidopsis*, major structural aspects of leaves are for the most part, unaltered (Cui et al., 2007). These reports also support the hypothesis that C_3_ plants may have functional repressors in the leaves that mediate the down-regulation of the key genes needed for Kranz and C_4_ differentiation irrespective of the amount of SCR or SHR protein present during development. Other cell types may share many of the developmental signaling cascades with the endodermis in the stems and petioles, but their tissue specificity may be controlled by other SHR/SCR interacting proteins that function in their respective feed-forward differentiation pathways. This may also explain why SHR and SCR are found in developing stomata and in the case of SCR, in the L1 layer of the shoot meristem (Wysocka-Diller et al., 2000; Kamiya et al., 2003; Lim et al., 2005), tissues not associated with the endodermis. This suggests that although SHR and SCR are essential for the patterning and formation of the endodermis and other cell types, they do not individually confer cell specificity or “identity.” Analogous to the ABCE model of floral development (Bowman et al., 2012), specific variants of the endodermis may be under the combinatorial control of multiple factors that form a functional protein complex that regulates differentiation. In other words, SHR and SCR are essential base, or “E” type (Bowman et al., 2012) functions in the endodermal developmental program.

If SHR and SCR are not direct targets for engineering Kranz-type C4, then what genes are? From a variety of published reports, the most likely candidates to function with SHR and SCR are the interacting proteins which include, but may not be restricted to, the indeterminate-domain family of transcription factors (IDDs; Levesque et al., 2006; Welch et al., 2007; Tanimoto et al., 2008; Ogasawara et al., 2011). Within the roots and stems, different combinations of these factors promote the formation of root and stem endodermal identity, quiescent cells, and stem cells. For example, in *Arabidopsis* roots a combination of AtIDD10 and AtIDD3 maintains stem cell identity (Welch et al., 2007). In stems, AtIDD15/SHOOTGRAVITROPISM5 (SGR5) functions with AtSHR and AtSCR to promote starch sheath identity (Tanimoto et al., 2008). But the most interesting and tantalizing evidence for the involvement of the IDDs in BS development comes from IDD over-expression studies. For example, when AtIDD8/*Nutcracker*, a target and interacting protein of AtSHR and AtSCR in the root endodermis (Levesque et al., 2006), was over-expressed in *Arabidopsis* (Seo et al., 2011), photosynthesis and plastid structures and were both dramatically altered. Most notable of these alterations is that M chloroplasts displayed reduced granal stacking – similar to what is seen in the BS of C_4_ plants (Levesque et al., 2006). This finding is reminiscent to the conversion of leaves into petals where chromoplasts developed instead of chloroplasts in the petaloid-like structures (Pelaz et al., 2001) showing that physiology can be controlled by developmental programming.

Only one of the IDD genes has been characterized in a C_4_ plant thus far. In maize, loss of function of *Indeterminate growth1* (*ZmID1*), the founding member of the gene family, results in altered growth and flowering time (Colasanti et al., 1998). Interestingly, *id1* mutants also have altered expression of many of the genes involved in C_4_ biochemistry, suggesting there may be a broader role for ID1 in leaf development and physiology (Coneva et al., 2007). *ZmID1* is only expressed at the base of developing leaves and decreases as the leaf matures, suggesting a role in leaf development (Wong and Colasanti, 2007). In this region it is expressed in all cells. Therefore, based on overlapping expression with both *ZmScr* and *ZmShr* genes, and the altered expression of C_4_-related genes in the mutant, it is likely that *ZmID1* plays a role in the development of the C_4_ pathway in maize. Many of the other IDD, SHR-like and SCR-like genes in maize are also expressed at the base of the developing leaf and have either BS- or M-specific expression patterns (Li et al., 2010), suggesting potential roles in establishing C_4_ BS or M cell identity and cell-specific organization of physiology. However, more research is needed to elucidate these proposed roles for *ZmID*1 and other IDD genes in either the SCR/SHR or C_4_ developmental pathways in leaves.

The IDD class of genes may also have the potential to act as negative regulators of endodermal development and identity. Recently it was found that some of the members of the IDD gene family contain ethylene-responsive element binding factor-associated amphiphilic repression (EAR) domains (Wu et al., 2013), which have been shown to act as strong transcriptional repressors. Might these be the factors that keep the endodermis/Kranz program suppressed in C_3_ leaves as hypothesized earlier? Overall, the published data on the IDD class of genes suggests they may play a significant role in Kranz-type C_4_ regulation and development, both as potential positive and negative regulators. However, much more research is needed to explore the hypotheses presented here.

## EVOLUTION OF KRANZ-TYPE C4 MECHANISM IN MONOCOTS: REVISITING THE PHYLLODE HYPOTHESIS

The emergence of C_4_ in monocots appears to be ancient, arising with the grasses and sedges as they began to diverge from the other monocots (Sage et al., 2011). Can the hypothesis stated above, that Kranz-type C_4_ is a synergistic interaction between the photosynthetic cells and the endodermis, also shed light on the evolution of C_4_ in grass leaves? In order to explore this question, it is essential to compare eudicot and monocot leaf blade anatomy. Most important is to recognize the theory that monocot’s leaves may not be true “leaf blade” tissue when compared to the eudicots (Arber, 1918; Kaplan, 1973).

**FIGURE 5:**
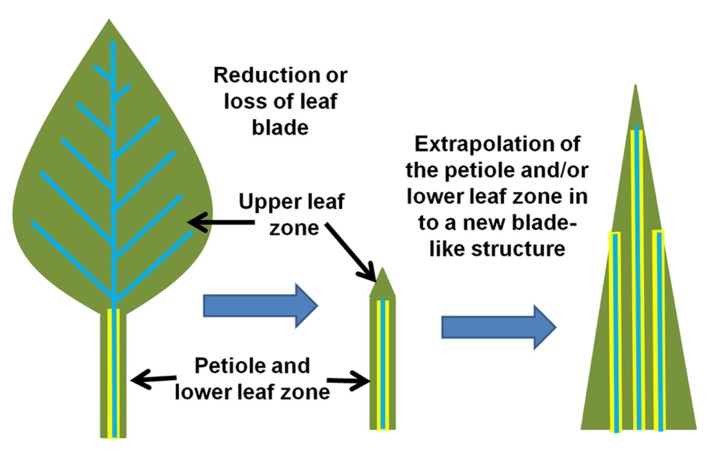
** Model for the evolution of the monocot leaf.** Two major morphological shifts may have occurred that dramatically altered the monocot lineage of plants. First, the reduction or loss of a canonical dicot leaf blade (depicted on the left) resulted in the petiole/lower leaf zone structure (center) that then assumed the role of the primary photosynthetic structure. Second, the petiole/lower leaf zone expanded and extrapolated into a new “leaf blade” (right) while also extending the endodermis/starch sheath into the new photosynthetic structure. This event may have also conditioned the parallel venation that is now associated with monocot leaves (model simplified and extrapolated from Arber, 1918; Kaplan, 1973; Nardmann et al., 2004).

It has been hypothesized that monocots evolved in an aquatic environment (Arber, 1918). This dramatically shifted the morphology of the shoot organs, such as leaves and stems. It is presumed that when these plants became submerged, their petioles or lower leaf blades became greatly extended in order to keep the leaf blades above or on the surface of the water. Over time, the upper leaf blade became greatly reduced, resulting in the petiole/lower leaf zone becoming the primary photosynthetic organ of the plant (Arber, 1918). The petiole/lower leaf zone then expanded and extrapolated into a new leaf blade (Tsiantis et al., 1999; Nardmann et al., 2004). The phyllode theory is illustrated in **Figure 5**. Whether the monocot leaf blade is derived from either the petiole as argued by Arber (1918) or the lower leaf zone (base including stipules) as argued by Kaplan (1973) is still unclear and highly debated. However, in either case, the extrapolation of either the lower leaf zone or the petiole into a new leaf base would support the arguments presented below.

**FIGURE 6:**
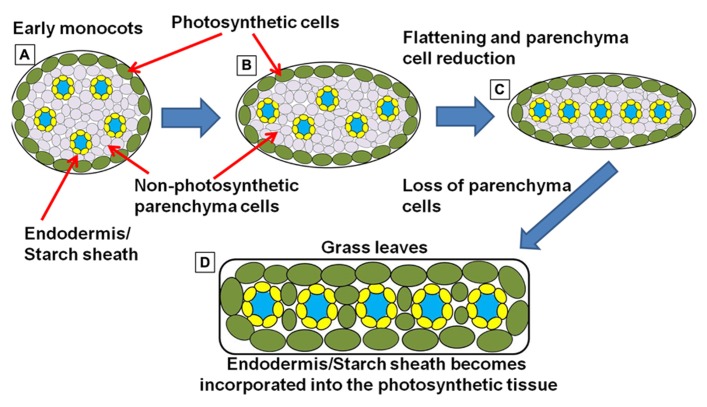
** Simplified schematic representation of cross sections through monocot “leaf blades” along the evolutionary trajectory toward the grasses.** **(A)** Simplified model of leaf structure in the early monocots (note: the early monocot leaves are depicted as radial structures in order to simplify the concepts presented). The vasculature encased in endodermal starch sheath tissue is separated from the outer photosynthetic layer by non-photosynthetic parenchyma cells. **(B)** The leaf structure begins to flatten and compress the vascular cores toward the center of the leaf, leading to a parallel vein patterning seen in **(C)**. In grasses and sedges **(D)** the non-photosynthetic parenchyma cells are reduced or completely absent, bringing the outer photosynthetic layers in contact with the endodermis/starch sheath layer that surrounds the vasculature (model simplified and extrapolated form Arber, 1918).

The reduction of loss of a true leaf blade still occurs in some dicots. For example, the amphibious plant *Ranunculus fluitans* has different phenotypes when plants develop in dry or submerged conditions (Burkhardt, 1977). When grown on dryer soil, the plants develop similar to normal eudicots. They have broad and fully expanded leaves and are compact. However, under submerged conditions, plants reduce or eliminate the upper leaf blade tissue, and extend and expand the petiole/lower leaf zone and stems into string-like structures (Osborne, 1984), similar to leaf structures in early monocots (Arber, 1918). Indeed, these plants show extensive plasticity in their ability to dramatically shift their shoot-specific morphology and physiology. In this submerged state, the stems and petioles take over the primary role of photosynthetic organ (Kutschera and Niklas, 2009). This raises the question: what if successive generations of such amphibious plants experience the flooded situation throughout the majority of their lifecycle? Could the plants permanently fix the flooded phenotype – leading to a grass like appearance due to the reduction of the upper leaf blade and an extrapolation of the petiole/lower leaf zone and the stem (**Figure 5**)? This morphological shift reduces many of the dicot leaf blade anatomical features, the most profound being the elimination of reticulate vein patterning. The formation of parallel veins in monocots is presumed to be derived from the merger of two sides of a previously radial-organized veins in the stems and petioles (**Figure 6**; Arber, 1918; Kaplan, 1973). Alternating phloem and xylem polarity within adjacent parallel veins of some of the monocot leaves supports this view of leaf blade evolution (Arber, 1918).

This may also explain why all of the C_4_ grasses and sedges use the Kranz-type mechanism (Langdale, 2011). As argued above, the petiole and lower leaf zone contains most, if not all, of the necessary anatomical and biochemical elements to establish the C_4_ photosynthetic syndrome (Hibberd and Quick, 2002; Brown et al., 2010; Slewinski et al., 2012). Thus, a new leaf structure extrapolated from this area of the leaf would inherently contain all of the necessary underlying components of Kranz-type C_4_.

However, most of the monocots utilize the C_3_ photosynthetic mechanism. Another look at monocot anatomy may explain why. In both C_3_ petioles/lower leaf blades and in early monocots, the endodermis and the outer layer of photosynthetic cells (beneath the epidermis) are usually separated by one or many layers of non-photosynthetic parenchyma cells (**Figure 6A**; Arber, 1918). These parenchyma cells block direct interaction between the starch sheath and the active site of photosynthesis. But as monocots evolved and the grass and sedge clade emerged, leaf structures become flattened and thinner (**Figures 6B,C**). The surrounding photosynthetic layers, one on either side of the leaf, start to invade the region of the central vascular strands (Arber, 1918), most likely through the progressive elimination of parenchyma cells. In many of the grasses and sedges, these parenchyma cells are entirely absent in the leaf blade and are usually only found in the large central mid-vein (**Figure 6D**). This anatomical adjustment would also bring the outer photosynthetic layer of cells in direct contact with the endodermis/starch sheath, allowing the two programs to interact. Thus, the morphological shifts that lead to the emergence of the grasses and sedges could also have been the events that pre-conditioned Kranz-type C_4_ within these clades.

**FIGURE 7:**
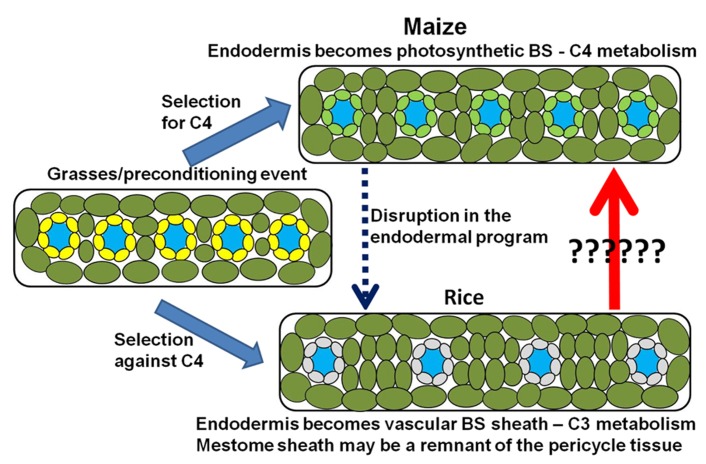
** Model for the photosynthetic divergence of rice and maize.** In maize (top), the endodermis/starch sheath becomes incorporated into the photosynthetic system – giving rise to the bundle sheath (Kranz anatomy) and synergistic interaction that underlies C_4_ photosynthesis. In rice (bottom), the synergistic interaction is selected against, thus maintaining a C_3_ photosynthetic mechanism. However, structural remnants of the endodermis/starch sheath remain after the endodermal program is either disrupted or suppressed. The endodermis/starch sheath becomes the non-photosynthetic vascular bundle sheath in rice, whereas remnants of the pericycle tissue may be adapted to form the mestome sheath layer within the vascular core.

This raises another important question. Why is rice C_3_ instead of C_4_? Under the phyllode theory of monocot evolution, the ancestors of rice may have been pre-conditioned for C_4_ metabolism in the same manner as other C_4_ grasses and sedges. However, it is important to remember that when compared to C_3_ photosynthesis, the C_4_ mechanism is energetically more expensive. It takes 18 ATP to fix one CO_2_ molecule in the C_3_ mechanism and 30 ATP in the C_4_ system (Langdale, 2011). It is possible that the C_4_ preconditioning event in the grasses did not confer an advantage within the environment in which the ancestors to domestic rice evolved. Thus the ancestors of rice and other C_3_ grasses may have either repressed or allowed the degradation of the C_4_ metabolic pathway in the leaf tissue. Under these assumptions, it can be argued that the vascular BS in rice may be a remnant of the endodermal tissue and the mestome sheath may be a remnant of the pericycle (**Figure 7**; Martins and Scatena, 2011). It is interesting to point out that it only took one mutation in the SCR gene of maize to produce many of the anatomical features that are seen in rice. Most notably, the starch-less BS cells reported in the *scarecrow* mutant of maize (Slewinski et al., 2012) have a striking resemblance to the vascular BS in rice (Langdale, 2011). Both cell types form a non-photosynthetic BS with undifferentiated plastids. Additionally, there are many other monocots that followed the same evolutionary trajectory as the grasses, producing flattened leaf blades that lack non-photosynthetic parenchyma cells, but retaining the C_3_ photosynthetic mechanism.

This may be why in oat-maize addition lines, the addition of individual maize chromosomes do not confer a functional C_4_ photosynthetic mechanism (Tolley et al., 2012), because the oat C_4_ program may have been suppressed or undergone degradation in leaves. However, in these studies, it is also important to take into consideration the caveats of the experiment itself. For example, in wheat, maize chromosomes are destroyed after fertilization (Lefebvre and Devaux, 1996), a phenomenon that is exploited in the production of double haploid wheat and oat. Thus, the addition of individual chromosomes may not represent a true “addition” of C_4_ genes that can be expected to function normally. The additional alien chromosome may undergo inactivation when taken out of the context of it native genomic and cellular context. This is commonly the case when exotic chromosomes are added into animal cell lines. It is likely, as mentioned above, the underlying factors that give rise to the endodermis and starch sheath, the SHR and SCR proteins, are already present within the nuclei of cells that comprise the C_3_ BS (Wysocka-Diller et al., 2000; Gardiner et al., 2011). Analogous to the C_3_ BS, it is possible that the negatively regulating interacting factors are in place within rice leaves – suppressing the development of full or sufficient endodermal identity. Suppression of the C_4_ pathway in hybrids between closely related C_3_ and C_4_ species has also been well documented (Brown and Bouton, 1993), supporting the hypothesis that C_3_ plants repress the activation of sufficient endodermal program in the leaves. Thus, if the full genome complement fails to activate a C_4_-like state in C_3_–C_4_ hybrids, it is unlikely that an individual chromosome, a partial genomic component, can initiate the C_4_ program within the C_3_ context.

The mechanism of C_4_ suppression or down-regulation could also be argued for many of the C_3_ grasses such as bamboo, oat, and wheat. Intriguingly, five independent reversals from C_4_ to C_3_ have been reported in the grasses (Vicentini et al., 2008). Is it possible that the ancestor at the base of the Pooideae (containing oat, barley, and wheat), Ehrhartoidea (containing rice), and Bambusoideae (containing bamboo) families underwent a C_4_ to C_3_ reversal early in its evolution? These three families contain only C_3_ species, unlike the majority of the other grass families such as Paniceae, Andropogoneae, and Centothecoideae in which some or all of its members contain C_4_ species (Vicentini et al., 2008; Sage et al., 2011). It is tempting to speculate that it is harder to re-evolve the C_4_ mechanism from a C_4_ to C_3_ reversal species than it is to newly evolve from a basic C_3_ species.

However, there does seem to be some hope for rice. When photosynthesis was surveyed in diverse rice species, considerable variation in photosynthetic rates was found (Yeo et al., 1994). None of the rice species were shown to employ the C_4_ mechanism, but some varieties had unusually low photorespiration rates, as well as increased phosphoenolpyruvate carboxylase activity and photosynthetic rates that are comparable to reported C_3_–C_4_ intermediate species. Thus, similar to the arguments for the origin of the vascular BS, some physiological aspects of the ancient C_4_ preconditioning event in the grasses may persist in a few rice species.

## FUTURE PERSPECTIVES

How can we transfer the Kranz-type C_4_ syndrome into C_3_ crops such as soybean and rice? From the hypothesis describe in this paper, the conversion of dicot species such as soybean may be easier than previously envisioned. Isolation of both the positive and negative regulators that control endodermal development would be the first step in engineering C_4_ by recapitulating evolution. In the case of C_4_ rice, the hypotheses and arguments made in this manuscript suggests that there may be alternative paths that might achieve this goal. Here I suggest that there might be two potential engineering trajectories. The first is to completely overhaul the physiology of the rice leaf with transgenic constructs that target the metabolism directly. The second is to try to reawaken the hypothesized C_4_-like state of rice’s distant past.

## MATERIALS AND METHODS

### PLANT MATERIALS, GROWTH CONDITIONS, AND TISSUE PREPARATION

Stocks containing the *lsn* mutation were kindly provided by Giuseppe Gavazzi at the Università degli Studi di Milano, Milan, Italy. Plants heterozygous for the mutation were self-pollinated to produce segregating families of mutant and wild type plants for analysis. *lsn* mutant and wild type plants were grown until the sixth leaf emerged. Leaves four and five were used for the analysis. Plants were grown and tissue processed, fixed, and stained for light and electron microscopy as described in Slewinski et al. (2012).

Maize lines containing the pin-formed1A-Yellow Fluorescent Protein (Pin1A-YFP) transgene were grown, prepared, and visualized as described in Slewinski et al. (2012).

## Conflict of Interest Statement

The author declares that the research was conducted in the absence of any commercial or financial relationships that could be construed as a potential conflict of interest.
